# Antagonism of Quorum Sensing Phenotypes by Analogs of the Marine Bacterial Secondary Metabolite 3-Methyl-*N*-(2′-Phenylethyl)-Butyramide

**DOI:** 10.3390/md17070389

**Published:** 2019-07-01

**Authors:** Susan M. Meschwitz, Margaret E. Teasdale, Ann Mozzer, Nicole Martin, Jiayuan Liu, Stephanie Forschner-Dancause, David C. Rowley

**Affiliations:** 1 Department of Chemistry, Salve Regina University, Newport, RI 02840, USA; 2 Department of Biomedical and Pharmaceutical Sciences, College of Pharmacy, University of Rhode Island, Kingston, RI 02881, USA

**Keywords:** quorum sensing, *Vibrio*, phenethylamide

## Abstract

Quorum sensing (QS) antagonists have been proposed as novel therapeutic agents to combat bacterial infections. We previously reported that the secondary metabolite 3-methyl-*N*-(2′-phenylethyl)-butyramide, produced by a marine bacterium identified as *Halobacillus* *salinus*, inhibits QS controlled phenotypes in multiple Gram-negative reporter strains. Here we report that *N*-phenethyl hexanamide, a structurally-related compound produced by the marine bacterium *Vibrio* *neptunius*, similarly demonstrates QS inhibitory properties. To more fully explore structure–activity relationships within this new class of QS inhibitors, a panel of twenty analogs was synthesized and biologically evaluated. Several compounds were identified with increased attenuation of QS-regulated phenotypes, most notably *N*-(4-fluorophenyl)-3-phenylpropanamide against the marine pathogen *Vibrio harveyi* (IC_50_ = 1.1 µM). These findings support the opportunity to further develop substituted phenethylamides as QS inhibitors.

## 1. Introduction

Bacterial populations synchronize gene expression via the release, detection, and biochemical response to small signaling molecules called autoinducers [1,2]. In the case of pathogenic bacteria, this chemical communication process, also referred to as quorum sensing (QS), coordinates phenotypes such as production of virulence factors, biofilms, and swarming motility [3,4,5,6,7]. Hence, interference with QS pathways provides an opportunity to attenuate pathogenicity, thereby representing a novel mechanism for battling bacterial infections [8,9,10,11]. Furthermore, several studies have demonstrated increased susceptibility of pathogenic bacteria to antibiotics when used in combination with QS inhibitors [12,13].

Natural product investigations have yielded structurally distinct quorum sensing inhibitors (QSIs), such as the brominated furanones isolated from the marine red alga *Delisea pulchra* [14,15] and the γ-lactone, plakofuranolactone, isolated from the marine sponge *Plakortis* cf. *lita* [16]. Marine organisms, including plants, animals, and microorganisms, have proven to be a particularly rich source of QSIs with diverse structures [17]. Moreover, synthetic modifications to many of these naturally-occurring scaffolds have led to higher affinity antagonists against bacteria pathogens [18]. For example, the marine honaucins, isolated from the bloom-forming cyanobacterium *Leptolyngbya crossbyana*, were found to inhibit QS signaling-dependent phenotypes in *V. harveyi* and an engineered *Escherichia coli* strain, and synthetic modifications resulted in improved QS inhibition as compared to the natural products [19].

Gram-negative bacteria commonly use *N*-acyl homoserine lactones (AHL) as signals, which bind their cognate receptor proteins to activate gene expression. These autoinducers share a conserved *L*-homoserine lactone moiety, while the length and sites of oxidation on the acyl chain dictate the species-specificity [20]. Antagonist activity can arise from simple structural variations to the native autoinducer [21,22,23]. For example, replacement of the lactone with a thiolactone in the native autoinducer can provide both improved potency and hydrolytic stability [24,25]. Modifying the length of the acyl chain can also impart antagonist activity, as demonstrated in the case of the plant pathogen *Agrobacterium tumefaciens* [26]. Previous studies also demonstrate that incorporation of aryl functionality with electron withdrawing groups onto the acyl side chain renders many AHL mimics as potent QSIs [27,28,29]. For example, termination of the acyl chain of the *Pseudomonas aeruginosa* autoinducer butanoyl-homoserine lactone with 4-bromophenyl interrupts AHL-mediated biofilm formation [30]. Hence, synthetic modifications to the natural substrates have proven to be useful in creating QSIs.

**Figure 1:**
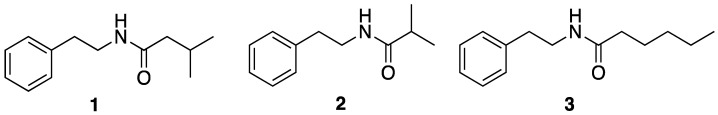
Chemical structures of phenethylamide natural products.

We previously reported that phenethylamide secondary metabolites (**1** and **2**, Figure 1), produced by marine *Halobacillus salinus* strain C42 obtained from the surface of a seagrass sample, inhibit QS regulated phenotypes in three Gram-negative reporter strains. Specifically, 3-methyl-*N*-(2′-phenylethyl)-butyramide (**1**) inhibits bioluminescence by the marine pathogen *V. harveyi*, violacein production by *Chromobacterium violaceum*, and green fluorescent protein (GFP) production by the QS sensor strain *Escherichia coli* JB525 [31]. The close congener 2-methyl-*N*-(2′-phenethyl)-propionamide (**2**) demonstrated reduced potencies against these three reporter strains. Here we report the identification of *N*-phenethyl hexanamide (**3**), produced by a *Vibrio neptunius* strain, as a closely related QSI. The variable potencies of these QSIs encouraged the synthesis of twenty analogs to help define structure–activity relationships (SAR), resulting in the identification of more potent compounds against these reporter strains.

## 2. Results

### 2.1. N-Phenethyl Hexanamide from Vibrio neptunius RIP07-147

Using our previously described cell–cell co-cultivation assay to identify marine bacteria with QSI potential against *V. harveyi* BB120 [17], we found that strain RIP07-147 (GenBank accession number MK821060), identified as a *V. neptunius* by 16S rRNA sequence comparison, demonstrated both antibiotic and bioluminescence inhibition activities. We were unaware of any previous natural product investigations of this species, and therefore undertook further study of this strain. RIP07-147 was cultivated on marine agar trays at 24 °C for 48 h. Following extraction of the whole cultures with ethyl acetate, bioassay-guided fractionation was pursued on the resulting extract using repeated reversed-phase chromatography, and bioactivity was followed by monitoring QS-controlled bioluminescence in the sensor strain *V. harveyi* BB120 as previously described [31]. These studies revealed that the hybrid PKS-NRPS secondary metabolite andrimid [32] was responsible for antibiotic activity, while QSI activity was due to *N*-phenethyl hexanamide (**3**) [33]. The structures were confirmed by comparison of ^1^H NMR and MS data with literature values, and later by synthesis in the case of **3** (see below).

### 2.2. Bioassay Testing

Compounds were tested in triplicate against three established QSI sensor strains [31]. *V. harveyi* causes disease in a variety of marine animals, especially shrimp [34], and has been previously used in the discovery of QSIs [35,36,37]. *V. harveyi* BB120 responds to the autoinducers 3-hydroxybutanoyl-L-homoserine lactone (HBHL) AI-1, the furanosyl borate diester AI-2, and (S)-3-hydroxytridecan-4-one (CAI-1) to regulate a variety of bacteria behaviors [38]. *C. violaceum* is a Gram-negative bacterium that produces violacein, an antibiotic purple pigment, under QS control using the autoinducer *N*-hexanoyl-L-homoserine lactone (HHL) [39]. Finally, the panel was screened for QSI activity using *E. coli* JB525, a mutant *E. coli* harboring the *gfp* plasmid pJBA132 linked to the LuxI/R quorum sensing system of *Vibrio fischeri*. This sensor strain produces an unstable green fluorescent protein (GFP) in response to exogenous C_6_-C_8_ AHL autoinducers [40]. Broth dilution assays with *E. coli* JB525 were conducted in the presence of 32 nM HHL, as we found this autoinducer provided the most consistent results and was used as a positive control in a similar *E. coli* reporter system [41].

Phenethylamide **3** inhibited *V. harveyi* bioluminescence (IC_50_ = 99 μM) and violacein production by *C. violaceum* (ZOI = 14 mm), but lacked activity against *E. coli* JB525, demonstrating that modest changes in the alkyl chain impacts the anti-QS activity (Table 1).

### 2.3. Analog Design and Biological Results

**Figure 2:**
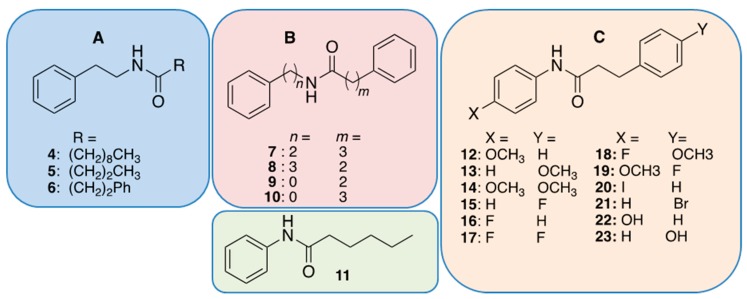
Chemical structures of synthetic analogs. Panel **A** compounds represent modifications to the acyl chain (R). Panel **B** compounds represent modifications to the chain length on either side of the of the amide bond. Panel **C** compounds represent modifications to the phenyl rings.

The structural simplicity of the phenethylamide QSIs, along with their variable activities against three different sensor strains, encouraged the preparation of synthetic analogs to explore structure–activity relationships. Specifically, a panel of analogs was designed to explore the effects of substitutions on the phenyl rings (X and Y), distance of the amide bond from the phenyl group (*n*), and chain length (*m*) and modifications (R) to the acyl group (Figure 2).

The initial analogs were designed to investigate modifications to the acyl chain (R) and included compounds **4**–**6** (Figure 2A). Prior SAR studies of HHL, the native autoinducer of *C. violaceum,* demonstrated that extending the length of the aliphatic chain (>C_10_) resulted in the creation of antagonists [42]. In compound **4**, extending R by four carbons (decanoyl) relative to **3** abolished activity against *C. violaceum* but was equipotent against *V. harveyi*. In compound **5**, installation of a shortened butanoyl chain had minimal effect on activity versus *C. violaceum* (ZOI = 21 mm) but abolished activity against *V. harveyi*. Interestingly, all modifications to the 3-methylbutanoyl chain of **1** had detrimental effects on the activity against *E. coli* JB525 (Table 1). These results demonstrate modifications to the acyl chain length can be used to tune the QSI to a particular QS system.

Previous studies aimed at designing QSIs demonstrate the benefit of installing a terminal phenyl ring on the AHL acyl side chain or as a replacement for the AHL lactone ring. For example, 4-phenylbutanoyl-homoserine lactone and 3-oxo-C_12_-2-aminophenol [23] were previously reported as potent Lux-R type antagonists [29]. With this in mind, compound **6** was synthesized and found to increase potency by nearly 6-fold against *V. harveyi* (IC_50_ = 17 μM) in comparison to **1**. However, compound **6** lacked activity against either *C. violaceum* or *E. coli* JB525.

The diphenyl motif was further expanded (Figure 2B, compounds **7**–**11**) by investigating modifications to the chain length on either side of the amide bond (*n* and *m*). Increasing the m linker by one carbon (m = 3) proved detrimental to the potency against *V. harveyi* (**7**, IC_50_ = 94 μM), while increasing *n* to three (**8**, IC_50_ = 29 μM) had a modest negative impact. Conversely, anilines (*n* = 0) resulted in much improved potency against *V. harveyi*. Compound **9** was 16-fold more active in *V. harveyi* versus the natural product **1**, 2-fold more active against *E. coli* JB52, and retained activity against *C. violaceum* (ZOI = 11 mm). Interestingly, compound **11**, which replaces the phenylethyl group of **9** with the pentyl chain of natural product **3**, demonstrated a 5-fold improved activity against *E. coli* JB525 (IC_50_ = 1.1 μg/mL).

We next turned our attention to installing substituents on the phenyl rings of **9** (Figure 2C). We noted that several previous reports demonstrated the benefit of electron withdrawing groups in a para position for improved QSI activity [21,22,28]. Initially, we investigated fluorine or a methoxy group in the para positions to compare the effects of an electron withdrawing and electron donating substituents (compounds **12**–**19**, Figure 2C). Our previous work on aryl beta-keto esters as QSIs of *V. harveyi* BB120 showed the most active derivatives to be 4-fluoro and 4-methoxy phenyl substituted analogs [35]. Here, the 4-fluoro derivative **16** resulted in nearly 6-fold improved inhibition against *V. harveyi* as compared to the non-substituted **9**, while a methoxy substituent (**12**) improved potency by less than 2-fold. Installing a fluorine (**15**) or methoxy (**18**) substituent in the para-position of the opposite phenyl ring resulted in only a 2-fold improvement versus *V. harveyi* as compared to **9**. Installing para-substituents on both phenyl rings had variable effects on potency (**14**, **17**–**19**), with the addition of a methoxy group to both phenyl rings (**14**) having a detrimental effect in all three assays, suggesting either an electronic or steric limitation.

Replacement of the fluorine on the amide phenyl ring with an iodine atom (**20**), or inclusion of a para-bromine atom on the acyl phenyl ring (**21**) had drastic effects, abrogating activity against all three sensor strains, suggesting either a steric or electronic effect [43]. Replacing the methoxy with a hydroxyl group on either phenyl ring (**22** and **23**) was also detrimental to the activity against *V. harveyi* but improved activity against *E. coli*, suggesting a variable hydrogen bonding role for the hydroxyl group in binding to certain Lux-R type receptors, such as the *V. fischeri* homolog in JB525.

**Figure 3:**
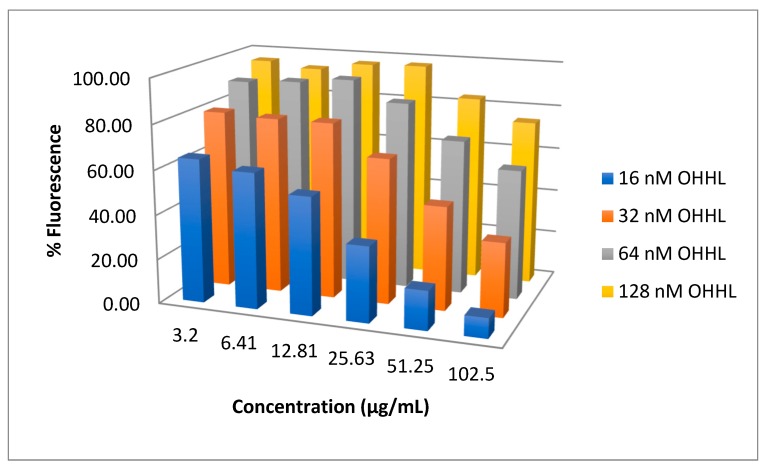
Bar graph showing green fluorescent protein (GFP) production (fluorescence) at various concentrations of antagonist (**11**) and agonist (OHHL). The inhibitory activity of **11** is surmounted by higher concentrations of OHHL agonist, consistent with an antagonist-agonist relationship. The concentration of **11** is in µg/mL and OHHL in nM. There were significant effects of compound **11** concentrations, OHHL concentrations and interactions (two-way ANOVA F_23,71_ = 342.3098, *p* < 0.0001, Supplementary Material Table S1). Standard deviation error bars are included in a 2D version of the graph (Supplementary Material Figure S5).

Compound **11** was the most potent inhibitor of the LuxR construct *E. coli* JB525 (IC_50_ = 1.1 μg/mL). To explore if **11** is a competitive antagonist of AHLs at the Lux R receptor, it was tested in serial dilutions against rising concentrations of *N*-(3-oxohexanoyl)-L-homoserine lactone (OHHL, 16–512 nM). We previously used this strategy to determine that **1** was a competitive antagonist of AHL mediated QS in *E. coli* JB525 [31]. Increased OHHL surmounted the inhibitory effects of **11** (Figure 3), consistent with an agonist-antagonist relationship, and suggesting a similar mechanism as **1** for inhibition of the LuxR controlled GFP expressed in *E. coli* JB525 [31].

Growth curves were conducted on all of the diphenyl analogs of compound **9** (compounds **12**–**19**, Figure 2C) and *N*-phenylhexanamide (**11**) at 100 μM, which is well above the IC_50_ value of the compounds, to ensure that the observed inhibition of luminescence was not due to inhibition or delay in growth of *V. harveyi* by the analogs. None of the compounds demonstrated a delay in growth (Supplementary Material Figures S1–S3). Additionally, a luminescence curve for compound **16**—the most active compound—demonstrated that the luminescence inhibition persists for the duration of luminescence production by the untreated bacteria (Supplementary Material Figure S4).

## 3. Discussion

*N*-phenethyl hexanamide (**3**) was isolated from a marine *V. neptunius* strain and identified as a QSI against the pathogen *V. harveyi*. To date, this is the first secondary metabolite reported for this bacteria species. *N*-phenethyl hexanamide adds to a small group of previously described phenethylamide QSIs produced by marine bacteria strains belonging to the genera *Halobacillus* [31,33] and *Oceanobacillus* [44], and bears structural resemblance to cyclic dipeptide QSIs comprising phenylalanine [45]. These natural products provided the motivation for synthesizing a panel of derivatives to more fully investigate this QSI class. The simple structures facilitated rapid assembly, frequently in one synthetic step with crystallization to prepare the desired pure product, further encouraging exploration of structure–activity relationships for these cell–cell signaling antagonists.

Many analogs were more active than the natural product **1** in the *V. harveyi* assay, with several compounds having IC_50_ values in the low micromolar range. In particular, diphenyl analogs appear to be the most promising QSIs against *V. harveyi*, and four of these (**9**, **13**, **16**, and **23**) demonstrated activity against all three reporter strains. While a general QSI might be desirable for treating infections caused by pathogens that use AHL-mediated signaling, it appears that the phenethylamide scaffold is more easily modified to optimize activity against specific QS systems.

Substitution of phenethylamine with aniline yielded *N*-phenylhexanamide (**11**), which was the only analog demonstrating equal or more potent activity than **1** against all three reporter strains. Other groups have identified non-natural modulators of AHL-based QS in which the native homoserine lactone moiety has been replaced with a phenyl ring, which suggests that the lactone moiety is not necessary for receptor binding. For example, Smith et al. demonstrated that 2-(3-oxo-C12-amino)phenol inhibits GFP production in a *Pseudomonas aeruginosa* strain constructed to express GFP in its LasR QS circuit [23].

Taken together, our results suggest that further exploration of the diphenyl analogs of the marine phenylethyl amide secondary metabolites (**1**–**3**) may yield more potent QSIs and highlights the need for increased investigation of marine microbes for the discovery and development of new anti-QS compounds.

## 4. Materials and Methods

### 4.1. Media

Bioassay media consisted of the following: (1) Marine broth (MB) containing 1 g yeast extract and 5 g peptone (Alpha Biosciences, Baltimore, MD, USA) per L synthetic seawater (Instant Ocean; 36 g per L); (2) Luria–Bertani broth containing 4 g sodium chloride (LB_4_); and (3) nutrient broth (NB) containing 5 g yeast extract and 10 g tryptone per L DI H_2_O. For agar media, 15 g agar per L of H_2_O was used.

### 4.2. Reporter Strains

*Vibrio harveyi* BB120 [3] a wild-type, bioluminescent strain, was cultivated at 30 °C in MB. *C. violaceum* ATCC 12472 was cultured at 29 °C with shaking in NB. *E. coli* JB525 is *E. coli* MT102 harboring the *gfp* plasmid pJBA132 and produces an unstable green GFP in response to C6-C8 AHL autoinducers [40]. *E. coli* JB525 was cultured in LB_4_ at 30 °C.

### 4.3. Isolation and Sequencing RIP07-147

The bacterial strain RIP07-147 (GenBank accession number MK821060) was isolated from a suspended marine particle collected in August 2007 in the East Passage of Narragansett Bay, Rhode Island, USA. The isolate was grown overnight at 24 °C in YP media and DNA was extracted using the DNeasy blood and tissue kit (Qiagen, Hilden, Germany) per the manufacturer’s protocol. PCR amplification of the bacterial 16S rRNA gene was accomplished using universal bacterial primers 27F and 1392R [46]. The isolate was identified based on 97.01% homology to *V. neptunius* strain LMG 20536 (22 February 2019) [47,48].

### 4.4. Co-Cultivation with V. harveyi BB120

An overnight culture of *V. harveyi* BB120 was diluted into 5 mL of molten MB soft agar at 40 °C and poured atop a MB agar plate. Two μL of an overnight culture of the test isolate, RIP07-147, was spotted onto the *V. harveyi* lawn. The plate was incubated at 24 °C overnight and imaged with a Typhoon 9410 variable mode imager (GE Healthcare Bio-Sciences, Piscataway, NJ, USA) in chemiluminescence mode. Zone of no light bioluminescence was measured to the nearest mm.

### 4.5. Isolation of N-Phenethyl Hexanamide

RIP07-147 was inoculated on yeast and peptone (YP) agar media in three 16 × 30 cm aluminum pans. The pans were incubated at 24 °C for 48 h. The agar was then extracted with ethyl acetate, filtered, and the liquid portion concentrated in vacuo. The extract was adsorbed onto C18 resin and fractionated by vacuum liquid chromatography with step-wise gradients of 100% water to 100% methanol (20% methanol increments) with a final acetone wash. Using the *V. harveyi BB120* assay (see below), the active constituent was determined to be in the 60% and 80% methanol fractions. These fractions were combined and further purified by HPLC (Waters Xterra RP_18_ 19 × 100 mm, 20 to 80% methanol over 30 min at 5 mL/min) to yield **3** as the single active compound. Compound **3** was identified as *N*-phenethyl hexanamide by comparison of ^1^H NMR spectroscopy and mass spectrometry data in comparison with literature data [33].

### 4.6. Bioassays

#### 4.6.1. *V. harveyi* BB120 Broth Dilution Assay

An overnight culture of *V. harveyi* BB120 in MB was diluted (OD_600_ = 0.1), and 200 μL of the diluted culture was added to 10 mL of MB. One μL of test compounds dissolved in DMSO at 50 mg/mL was added to a 96-well clear bottom, white microtiter plate (Corning, 0.5% DMSO final concentration). The diluted cell culture was added to the wells of the opaque microtiter plate and incubated at 30 °C with shaking for 5 h. The plates were read on a SpectraMax Multimode Microplate Reader (Molecular Devices, Sunnyvale, CA, USA). Relative luminescence units (RLU) were normalized by the OD_600_ values. Percent luminescence was calculated by defining the untreated cells (no compound) as 100%.

#### 4.6.2. *C. violaceum* Disc Diffusion Assay

Disc diffusion assays were performed with pure compounds at 500 μg/disc. One hundred microliters of overnight bacterial culture were added to 10 mL of NB, vortexed, and then 100 μL of the diluted culture was spread atop an NB agar plate. Impregnated, sterile discs (6 mm) were laid onto the test plates and incubated overnight. Zones of inhibition (ZOI), as indicated by lack of pigment production, were measured to the nearest mm.

#### 4.6.3. *E. coli* JB525 Bioassay

Inhibition of fluorescence was determined using a method modified from Teasdale et al. [31]. Briefly, an overnight culture of *E. coli* JB525 in LB_4_ broth was diluted (OD_450_ 0.25) with fresh media. Cultures were treated with 32 nM HHL and test compound ranging from 2 to 250 μg/mL (0.5% final DMSO concentration in 200 μL) in a 96-well clear bottom, black microtiter plate. To determine antagonist–agonist relationships, each serial dilution of test compound (8–1000 μM) was challenged with each increasing OHHL concentration (16–512 nM) in three biologically separate replicates (0.7% DMSO final concentration). Plates were incubated with shaking at 30 °C for 3 h. Fluorescence was detected with an excitation at 475 nm and emission at 515 nm on the SpectraMax i3 multi-mode microplate reader (Molecular Devices, Sunnyvale, CA, USA). Growth was evaluated after 3 h by optical density at 450 nm. Fluorescence values were normalized by optical density. For IC_50_ determination at 32 nM HHL, percent fluorescence was determined by defining control wells with 32 nM HHL as 100% fluorescence.

#### 4.6.4. Statistical Analysis

Assays were performed in biological and technical triplicate. Data were analyzed using GraphPad Prism 7. IC_50_ values were calculated using non-linear regression analysis and the values of each trial were averaged for the final reported value.

### 4.7. Chemical Syntheses

^1^H NMR spectra were recorded on a Bruker Avance (300 MHz) or a Bruker Biospin (400 MHz) spectrometer and mass spectra were recorded on a SCIEX QTOF 4600 using flow injection in 75% aqueous CH_3_OH containing 0.1% HCOOH. All reagents and compounds were purchased from Sigma-Aldrich or Acros Chemicals. Purification of the desired products was accomplished by either recrystallization (ethyl acetate and hexane), automated column chromatography on silica (CombiFlash, Teledyne Isco, Lincoln, NE, USA) using a linear gradient of hexanes in ethyl acetate (0%–100%), or by reverse-phase HPLC (Waters X-Terra Prep RP_18_ column, 19 × 100 mm, gradient of MeOH in H_2_O (0.1% formic acid, 5 mL/min). HPLC was performed on a Waters 600 with a 2487 dual wavelength detector set to λ 220 nm and λ 254 nm. Compounds were synthesized as follows.

#### 4.7.1. General Procedure for Coupling Reactions

The appropriate carboxylic acid in 50 mL acetonitrile was treated with HBTU (*N,N,N′,N′*-Tetramethyl-*O-*(1*H*-benzotriazol-1-yl)uranium hexafluorophosphate) (1.2 eq), diisopropylethyamine (1.5 eq), and the requisite amine. The reaction was stirred overnight at ambient temperature, concentrated in vacuo, and then partitioned between ethyl acetate and 0.1 M HCl. The organic phase was separated, sequentially washed with saturated sodium bicarbonate and water, dried over anhydrous sodium sulfate, filtered and concentrated in vacuo. The resulting products were purified by either crystallization or chromatography as described. Percent yields ranged from 30%–89%.

#### 4.7.2. Synthesis and Characterization of Compounds **1**–**23**

*3-Methyl-N-(2′-phenylethyl)-butyramide* (**1**). Iso-valeric acid and phenethylamine. Desired product was purified by crystallization (white crystals). ESI-MS [M + H]^+^ = 206.15; ^1^H NMR (400 MHz, CDCl_3_): δ 0.92 (d, *J* = 8.0 Hz, 6H), 1.98 (d, *J* = 8.0 Hz, 2H), 2.07 (m, 1H), 2.82 (t, *J* = 6.8 Hz, 2H), 3.53 (m, 2H), 5.46 (s, 1H) 7.15–7.35 (m, 5H).

*N-Phenethylhexanamide* (**3**). Hexanoic acid (4 mmol, 1 eq) and phenethylamine (4 mmol, 1 eq). Desired product was purified by HPLC (50%–75% MeOH in H_2_O over 10 min, white solid). ESI-MS [M + H]^+^ = 220.17; ^1^H NMR (400 MHz, CDCl_3_): δ 0.88 (t, *J* = 7.0 Hz, 3H), 1.29 (m, 4H), 1.59 (m, 2H), 2.09 (t, *J* = 7.5 Hz, 2H), 2.82 (t, *J* = 7.0 HZ, 2H), 3.52 (m, 2H), 5.44 (s, 1H), 7.19 (m, 5H).

*N-Phenethyldecanamide* (**4**). Decanoic acid (5 mmol, 1 eq) and phenethylamine (5 mmol, 1 eq). Desired product was purified by crystallization (white crystals). ESI-MS [M + H]^+^ = 276.12; ^1^H NMR (400 MHz, CDCl_3_): δ 0.88 (t, *J* = 8 Hz, 3H), 1.26 (s, 12H), 1.58 (t, *J* = 8 Hz, 2H), 2.11 (t, *J* = 8 Hz, 2H), 2.81 (t, *J* = 8 Hz, 2H), 3.51 (dt, *J* = 8 Hz, 2H), 5.61 (s, 1H), 7.18–7.32 (m, 5H).

*N-Phenethylbutryamide* (**5**). Butyric acid (4 mmol, 1 eq) and phenethylamine (4 mmol, 1 eq). Desired product was purified by HPLC (50% MeOH in H_2_O to 75% MeOH over 10 min, white solid). ESI-MS [M + H]^+^ = 192.06; ^1^H NMR (400 MHz, CDCl_3_): δ 0.91 (t, *J* = 7.5 Hz, 3H), 1.61 (m, 2H), 2.10 (t, *J* = 7.5 Hz, 2H), 2.81 (t, *J* = 7.5 Hz, 2H), 3.51 (m, 2H), 5.54 (s, 1H), 7.18–7.33 (m, 5H).

*N-Phenethyl-3-phenylpropanamide* (**6**). Hydrocinnamic acid (4 mmol, 1 eq) and phenethylamine (4 mmol, 1 eq). Desired product was purified by crystallization (white crystals). ESI-MS [M + H]^+^ = 254.17; ^1^H NMR (400 MHz, CDCl_3_): δ 2.43 (t, *J* = 6.8 Hz, 2H), 2.74 (t, *J* = 6.8 Hz, 2H), 2.95 (t, *J* = 6.8 Hz 2H), 3.48 (m, 2H), 5.42 (s, 1H), 7.04–7.31 (m, 10H).

*N-Phenethyl-4-phenylbutanamide* (**7**). 4-phenyl-butyric acid (5 mmol, 1 eq) and phenethylamine (5 mmol, 1 eq). Desired product was purified by crystallization (white crystals). ESI-MS [M + H]^+^ = 268.18; ^1^H NMR (400 MHz, CDCl_3_): δ 1.95 (m, 2H), 2.13 (t, *J* = 7.0 Hz, 2H), 2.63 (t, *J* = 8.0 Hz, 2H), 2.82 (t, *J* = 8.0 Hz, 2H), 5.46 (s, 1H), 7.14–7.33 (m, 10H).

*3-Phenyl-N-(3-phenylpropyl) propanamide* (**8**). Hydrocinnamic acid (4 mmol, 1 eq) and 3-phenyl-1-propylamine (4 mmol, 1 eq). Desired product was purified by HPLC (50% MeOH in H_2_O to 75% MeOH over 10 min, white solid). ESI-MS [M+Na]^+^ = 290.03; ^1^H NMR (400 MHz, CDCl_3_): δ 1.77 (m, 2H), 2.43 (t, *J* = 8Hz, 2H), 2.57 (t, *J* = 8Hz, 2H), 2.95 (t, *J* = 8Hz, 2H), 3.25 (t, *J* = 8Hz, 2H), 5.32 (s, 1H), 7.13–7.30 (m, 10H).

*N,3-Diphenylpropanamide* (**9**). Hydrocinnamic acid (4 mmol, 1 eq) and aniline (4 mmol, 1 eq). Desired product was purified by crystallization (white crystals). ESI-MS [M + H]^+^ = 226.04; ^1^H NMR (400 MHz, CDCl_3_): δ 2.66 (t, *J* = 8 Hz, 2H), 3.05 (t, *J* = 8 Hz, 2H), 7.10 (t, *J* = 8 Hz, 1H), 7.22–7.45 (m, 10H).

*N,4-Diphenylbutanamide* (**10**). 4-phenyl-butyric acid (4 mmol, 1 eq) and aniline (4 mmol, 1 eq). Desired product was purified by crystallization (white crystals). ESI-MS [M + H]^+^ = 240.04; ^1^H NMR (400 MHz, CDCl_3_): δ 2.08 (m, 2H), 2.35 (t, *J* = 8 Hz, 2H), 2.72 (t, *J* = 8 Hz, 2H), 7.11 (t, *J* = 8Hz, 1H), 7.21–7.34 (m, 8H), 7.51 (d, J = 8 Hz, 2H).

*N-Phenylhexanamide* (**11**). Hexanoic acid (4 mmol, 1 eq) and aniline (4 mmol, 1 eq). Desired product was purified by crystallization (white crystals, 89%). ESI-MS [M + H]^+^ = 192.14; ^1^H NMR (400 MHz, CDCl_3_): δ 0.91 (t, *J* = 6.5 Hz, 3H), 1.35 (m, 4H), 1.73 (m, 2H), 2.35 (t, *J* = 7.5 Hz, 2H), 7.26 (s, 1H), 7.10 (t, *J* = 7.3 Hz, 1H), 7.31 (t, *J* = 8.0 Hz, 2H), 7.52 (d, *J* = 7.8 Hz, 2H).

*N-4-Methoxyphenyl-3-phenylpropanamide* (**12**). Hydrocinnamic acid and *p*-anisidine. Desired product was purified by crystallization (pale purple crystals, 30%). ESI-MS [M + H]^+^ = 256.14; ^1^H NMR (300 MHz, CDCl_3_): δ 2.61 (t, *J* = 7.6 Hz, 2H), 3.75 (s, 3H), 3.02 (t, *J* = 7.7 Hz, 2H), 6.8 (d, *J* = 9Hz, 2H), 7.19–7.32 (m, 7H), 7.33 (bs, 1H).

*N-Phenyl-3-(4-methoxyphenyl)-propanamide* (**13**). 3-(4-methoxyphenyl) propanoic acid and aniline. Desired product was purified by crystallization (white crystals, 31%). ESI-MS [M+Na]^+^= 278.12; ^1^H NMR (300 MHz, CDCl_3_): δ 2.59 (t, *J* = 7.6 Hz, 2H), 2.95 (t, *J* = 7.6 Hz, 2H), 3.76 (s, 3H), 6.80 (d, *J* = 8.6 Hz, 2H), 7.13 (m, 3H), 7.26 (t, *J* = 7.0 Hz, 2H), 7.44 (d, *J* = 7.7 Hz, 3H).

*N-4-Methoxyphenyl-3-(4-methoxyphenyl)-propanamide* (**14**). 3-(4-methoxyphenyl) propanoic acid and *p*-anisidine. Desired product was purified by crystallization (white crystals, 78%). ESI-MS [M+Na]^+^ = 308.13; ^1^H NMR (300 MHz, CDCl_3_): δ 2.58 (t, *J* = 7.7 Hz, 2H), 2.95 (t, *J* = 7.6 Hz, 2H), 3.77 (s, 6H), 6.82 (d, *J* = 8.6Hz, 4H), 7.14 (d, *J* = 8.4 Hz, 2H), 7.34 (bs, 1H), 7.37 (d, *J* = 8.8 Hz, 2H).

*N-Phenyl-3-(4-fluorophenyl) propanamide* (**15**). 3-(4-fluorophenyl) propanoic acid and Aniline. Desired product was purified by crystallization (white crystals, 51%). ESI-MS [M+Na]^+i^ = 266.09; ^1^H NMR (300 MHz, CDCl_3_): δ 2.61 (t, *J* = 7.5 Hz, 2H), 3.00 (t, *J* = 7.5 Hz, 2H), 6.96 (t, *J* = 8.7 Hz, 2H), 7.13 (m, 2H), 7.28 (t, *J* = 7.9 Hz, 2H), 7.27 (d, *J* = 8.01 Hz, 2H), 7.44 (d, *J* = 7.8 Hz, 2H).

*N-(4-Fluorophenyl)-3-phenylpropanamide* (**16**). 4-fluoroaniline and hydrocinnamic acid. Desired products were purified by crystallization (white crystals, 42%). ESI-MS [M+Na]^+^ = 266.09; ^1^H NMR (300 MHz, CDCl_3_): δ 2.62 (t, *J* = 7.6 Hz, 2H), 3.01 (t, *J* = 7.6 Hz, 2H), 6.94 (t, *J* = 8.7 Hz, 2H), 7.17–7.37 (m, 7H), 7.43 (bs, 1H).

*N-4-Fluorophenyl-3-(4-fluorophenyl) propanamide* (**17**). 3-(4-fluorophenyl) propanoic acid and 4-fluoroaniline. Desired product was purified by crystallization (white crystals, 28%). ESI-MS [M+Na]^+^ = 284.09; ^1^H NMR (300 MHz, CD_3_OD) δ 2.63 (t, *J* = 8.5 Hz, 2H), 2.95 (t, *J* = 7.6 Hz, 2H), 6.99 (m, 5H), 7.24 (dd, *J* = 8.6 Hz, 5.5 Hz, 2H), 7.49 (dd, *J* = 9.2 Hz, 4.9 Hz, 2H).

*N-(4-Fluorophenyl)-3-(4-methoxyphenyl) propanamide* (**18**). 3-(4-methoxyphenyl)propanoic acid and 4-fluoroaniline. Desired product was purified by crystallization (white crystals, 39%). ESI-MS [M+Na]^+^ = 296.11; ^1^H NMR (300 MHz, CD_3_OD): δ 2.63 (t, *J* = 7.6 Hz, 2H), 2.92 (t, *J* = 7.7 Hz, 2H), 3.74 (s, 3H), 6.82 (d, *J* = 8.7 Hz, 1H), 7.04 (t, *J* = 8.9Hz, 2H), 7.17] (d, *J* = 8.9 Hz, 1H), 7.65 (d, *J* = 9.2 Hz, 2H), 7.65 (d, *J* = 9.2 Hz, 2H), 7.69 (bs, 1H).

*N-(4-Methoxyphenyl)-3-(4-fluorophenyl) propanamide* (**19**). 3-(4-fluorophenyl) propanoic acid and *p*-anisidine. Desired product was purified by crystallization (pale purple crystals, 62%). ESI-MS [M+Na]^+^ = 296.11; ^1^H NMR (300 MHz, CDCl_3_): δ 2.63 (t, *J* = 7.6 Hz, 2H), 2.97 (t, *J* = 7.6 Hz, 2H), 3.75 (s, 3H), 6.84 (d, *J* = 9.0 Hz, 2H), 7.02 (t, *J* = 8.8 Hz, 2H), 7.28 (d, *J* = 8.4 Hz, 1H), 7.29 (d, *J* = 8.4 Hz, 1H), 7.53 (d, *J* = 9 Hz, 2H), 8.98 (bs, 1H).

*N-(4-Iodophenyl)-3-phenylpropanamide* (**20**). 4-Iodoaniline and hydrocinnamic acid. Desired product was purified by crystallization (pale purple crystals, 40%). ESI-MS [M+Na]^+^ = 374.00; ^1^H NMR (300 MHz, CDCl_3_): δ 2.65 (t, *J* = 7.5 Hz, 2H), 3.05 (t, *J* = 7.5 Hz, 2H),6.92 (bs, 1H), 7.26 (m, 7H), 7.59 (d, *J* = 8.8 Hz, 2H).

*3-(4-Bromophenyl)-N-phenylpropanamide* (**21**). 3-(4-bromophenyl) propionic acid (4 mmol, 1 eq) and aniline (4 mmol, 1 eq). Desired product purified by crystallization. ESI-MS [M+Na]^+^ = 325.88; ^1^H NMR (400 MHz, CDCl_3_): δ 2.63 (t, *J* = 7.5 Hz, 2H), 3.03 (t, *J* = 7.5 Hz, 2H), 7.10 (m, 4H), 7.26 (d, *J* = 8 Hz, 1H), 7.32 (t, *J* = 7 Hz, 2H), 7.44 (m, 3H).

*3-(4-Hydroxyphenyl)-N-phenylpropanamide* (**22**). Compound **12** (1 mmol) was dissolved in 2 mL DMF, treated with iodocyclohexane (10 mmol), and refluxed under nitrogen for 14 h. The reaction was then cooled, poured into water (20 mL), and extracted with ethyl acetate (3 × 20 mL). The organic layer was washed sequentially with saturated aq. NaHSO_3_ and brine, dried over Na_2_SO_4_, filtered, and concentrated. The crude product was purified by column chromatography (white solid, 54%). ESI-MS [M+Na]^+^ = 264.10; ^1^H NMR (300 MHz, CD_3_OD): δ 2.61 (t, *J* = 7.0 Hz, 2H), 2.97 (t, *J* = 7.0 Hz, 2H), 8.9 (t, *J* = 7.7 Hz, 2H), 6.70 (d, *J* = 8.9 Hz, 2H), 7.25 (m, 5H), 7.25 (d, *J* = 9.0 Hz, 2H).

*N-Phenyl-3-hydroxyphenylpropanamide* (**23**). Prepared from **13** using identical method as for compound **22** and purified by column chromatography (white solid, 53%). ESI-MS [M + H]^+^ = 242.12; ^1^H NMR (300 MHz, CDCl_3_): d 2.62 (t, *J* = 7.6 Hz, 2H), 2.89 (t, *J* = 7.7 Hz, 2H), 6.75 (t, *J* = 8.6 Hz, 2H), 7.06 (m, 1H), 7.08 (d, *J* = 8.6 Hz, 2H), 7.27 (t, *J* = 7.9 Hz, 2 H), 7.64 (d, *J* = 7.6 Hz, 2H), 9.12 (bs, 1H).

## Figures and Tables

**Table 1:** Activity of natural products and their analogs against three reporter strains.

Compound	*V. harveyi* BB120		*E. coli* JB525		*C. violaceum* ATCC 12472
	IC_50_ (μM)	Std. Dev.	IC_50_ (μg/mL)	Std. Dev.	Zone of Inhibition ^a^ (mm)
1	110	12	11	3.5	20
2	NA		NA		12
3	99	5.9	NA		14
4	89	13	NA		NA
5	NA		NA		21
6	17	2.9	NA		NA
7	94	7.0	NA		NA
8	29	3.0	NA		NA
9	6.2	0.40	5.2	1.0	11
10	15		NA		NA
11	48	7.6	1.1	0.36	22
12	3.3	1.9	>200		12
13	5.6	3.6	32	12	9
14	86	2.7	>200		NA
15	3.5	1.6	3.8	1.0	NA
16	1.1	0.60	25	13	9
17	3.0	0.37	69	14	NA
18	6.0	2.0	NA		NA
19	12	8.1	>150		NA
20	NA		NA		NA
21	>200		NA		NA
22	19	1.2	56	2.2	13 ^b^
23	82	17	13	4.0	11

IC_50_ values greater than 500 were considered inactive and are designated as NA (no activity). ^a^ Zones of inhibition determined for 500 μg/disc. ^b^ Includes 8 mm zone of growth inhibition followed by zone of no violacein production.

## Supplementary Materials

The following are available online at https://www.mdpi.com/1660-3397/17/7/389/s1, Figures S1–S3: 24-h growth curves of V. harveyi in the presence of phenethylamide analogues, Figure S4: 24-h luminescence curves by *V. harveyi* BB120 in the presence of phenethylamide analogues **14** (100 μM) and **16** (10 µM) at concentrations that are above their IC_50_ values. Figure S5: 2D version of Figure 3 with error bars added. Bar graph showing GFP production (fluorescence) at various concentrations of antagonist (11) and agonist (OHHL). Error bars reflect at least three experiments each done in triplicates. Table S1: One-way ANOVA for effect of Compound 11 concentration.
