# Supplementary material for: Antagonism of Quorum Sensing Phenotypes by Analogs of the Marine Bacterial Secondary Metabolite 3-Methyl-N-(2′-Phenylethyl)-Butyramide
Source: Mar Drugs. 2019 Jul 1;17(7):389. doi: 10.3390/md17070389 (PMC6669561; doi:10.3390/md17070389)
Supplement: Supplementary file 1 [file marinedrugs-17-00389-s001.pdf]

Supplementary Information

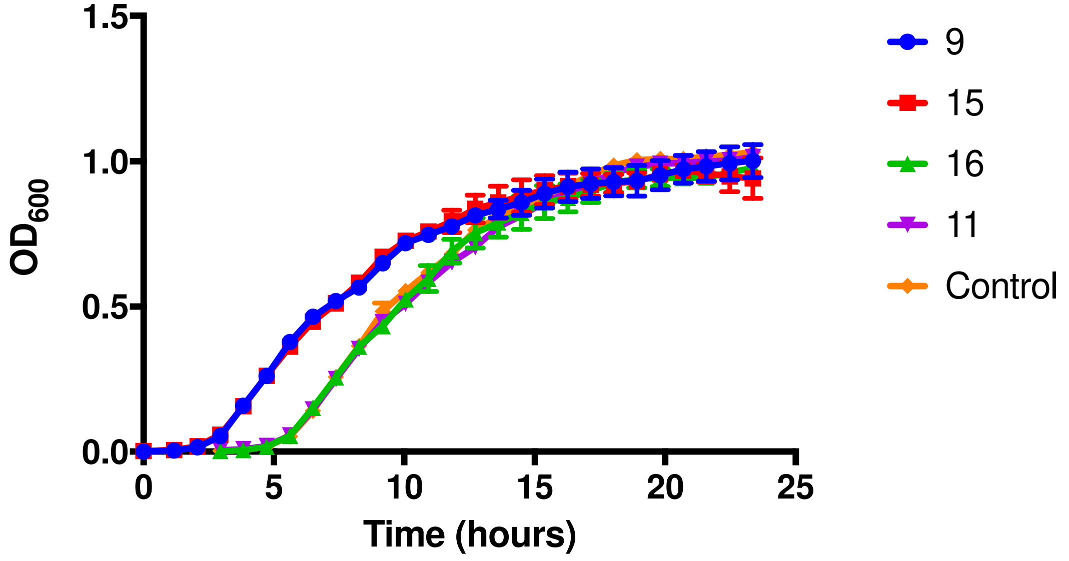

Figure S1. Growth of *V. harveyi* BB120 monitored by optical density at 600 nm for 24 hours. Cultures were treated with phenethylamide analogs **9**, **11**, **15**, and **16** at 100 μM, which is well above the IC_{50} value of the compounds. Control contains same concentration of DMSO as treatment but with no added compound. Error bars represent standard deviation of replicates.

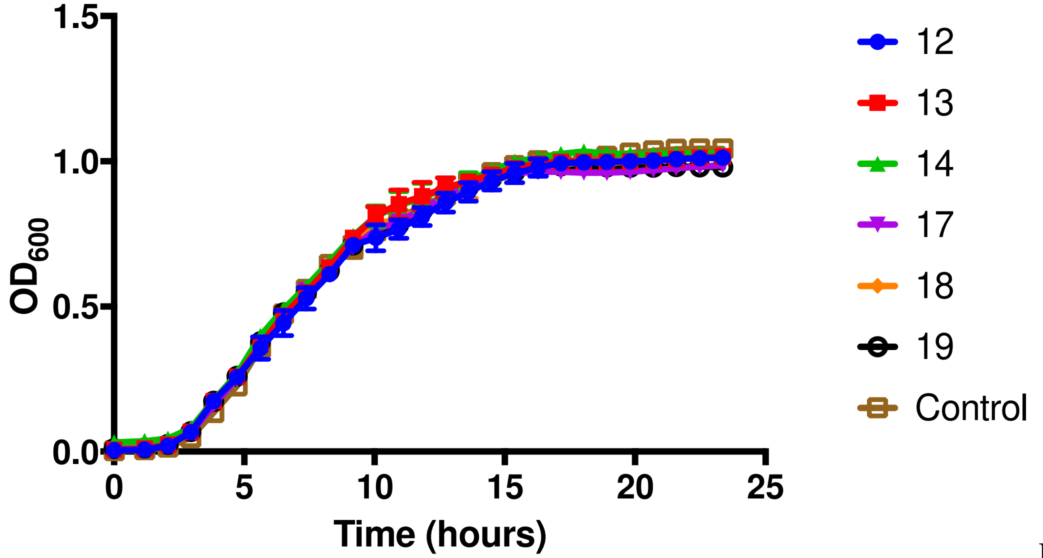

Figure

Figure S2. Growth of *V. harveyi* BB120 monitored by optical density at 600 nm for 24 hours. Cultures were treated with phenethylamide analogs **12-14** and **17-19** at 100 μM, which is well above the IC_{50} value of the compounds. Control contains same concentration of DMSO as treatment but with no added compound. Error bars represent standard deviation of replicates.

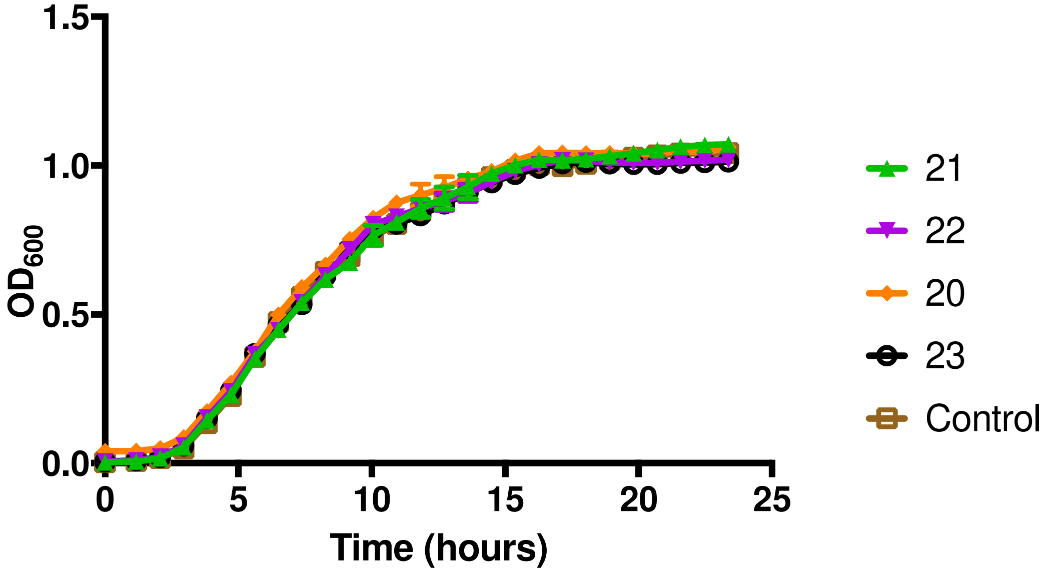

Figure S3. Growth of *V. harveyi* BB120 monitored by optical density at 600 nm for 24 hours. Cultures were treated with phenethylamide analogs **20-23** at 100 µM, which is well above the IC_{50} value of the compounds. Control contains same concentration of DMSO as treatment but with no added compound. Error bars represent standard deviation of replicates.

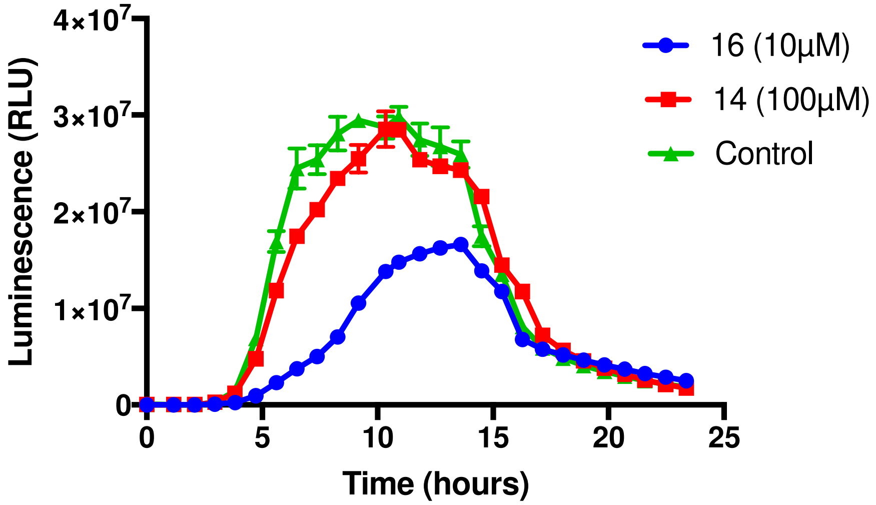

Figure S4. Luminescence by *V. harveyi* BB120 in the presence of phenethylamide analogues **14** (100 µM) and **16** (10 µM) at concentrations that are above their IC_{50} values**.** Control contains same concentration of DMSO as treatment but with no added compound. Error bars represent standard deviation of replicates.

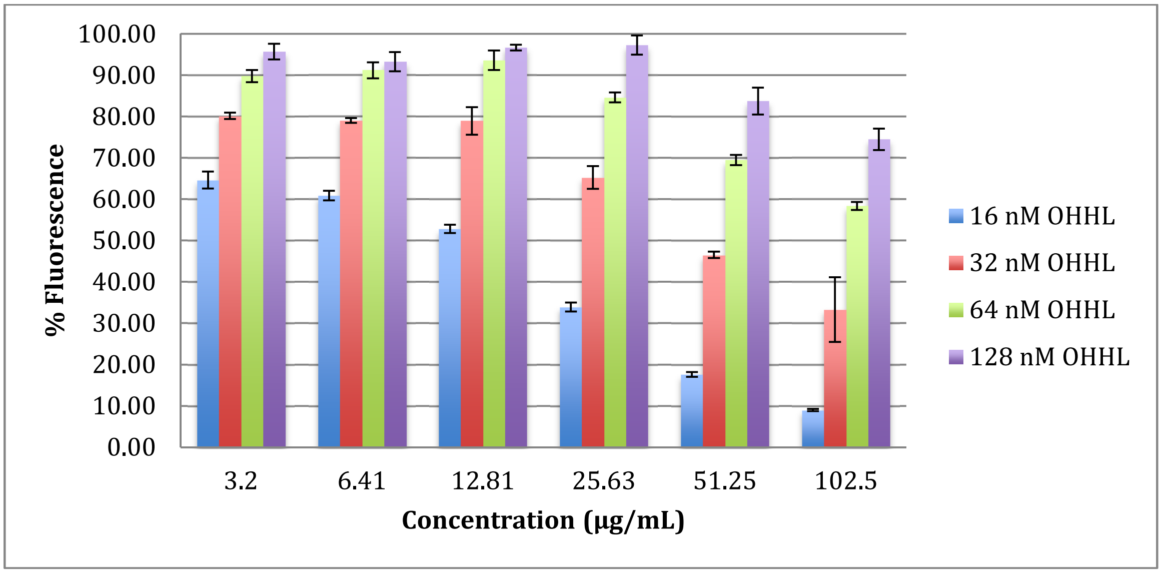

Figure S5. 2D version of Fig. 3 with error bars added. Bar graph showing GFP production (fluorescence) at various concentrations of antagonist (11) and agonist (OHHL). Error bars reflect at least three experiments each done in triplicates.

Table S1. One-way ANOVA for effect of Compound **11** concentration

One-Way ANOVA by OHHL Conc.				Post-hoc Tukey HSD test, significant differences (P<0.05) indicated by different letters					
	F	df	P	3.2 ug/ml	6.4 ug/ml	12.8 ug/ml	25.2 ug/ml	51.2 ug/ml	102.5 ug/ml
16 nM	1173.466	5, 17	<0.0001	A	B	C	D	E	F
32 nM	87.0107	5, 17	<0.0001	A	A	A	B	C	D
64 nM	235.7349	5, 17	<0.0001	A	A	A	B	C	D
128 nM	47.2729	5, 17	<0.0001	A	A	A	A	B	C

One-Way ANOVA by Cpd **11** conc.				Post-hoc Tukey HSD test, significant differences (P<0.05) indicated by different letters			
	F	df	P	128 nM	64 nM	32 nM	16 nM
3.2 ug/ml	209.4574	3, 11	<0.0001	A	B	C	D
6.4 ug/ml	246.2852	3, 11	<0.0001	A	A	B	C
12.8 nM	265.0294	3, 11	<0.0001	A	A	B	C
25.6 nM	589.1636	3, 11	<0.0001	A	B	C	D
51.2 nM	771.9131	3, 11	<0.0001	A	B	C	D
102.5 nM	144.1150	3, 11	<0.0001	A	B	C	D
